# Ferroptosis regulators, especially SQLE, play an important role in prognosis, progression and immune environment of breast cancer

**DOI:** 10.1186/s12885-021-08892-4

**Published:** 2021-10-29

**Authors:** Wenqing Tang, Fangshi Xu, Meng Zhao, Shuqun Zhang

**Affiliations:** 1 Department of Medicine, Xi’an Jiaotong University, Xi’an, 710061 Shaanxi China; 2 Department of Oncology, The Second Affiliated Hospital of Xi’an Jiaotong University, No. 157, West Five Road, Xi’an, 710000 Shaanxi China

**Keywords:** Breast cancer, Ferroptosis, Risk signature, Prognosis, Immune checkpoint inhibitors, SQLE

## Abstract

**Background:**

Ferroptosis, a new form of programmed cell death, has great potential for cancer treatment. However, the roles of ferroptosis-related (FR) genes in breast cancer (BC) remain elusive.

**Materials and methods:**

Using TCGA database, a novel FR risk signature was constructed through the Lasso regression analysis. Meanwhile, its prognostic value was assessed by a series of survival analyses. Besides, a nomogram was constructed to predict the overall survival rate (OSR) of individual at 1,3,5 year. Four validation cohorts (*n* = 2248), including METABRIC, GSE58812, GSE20685 and ICGC-KR datasets, were employed to test the prognostic value of FR risk signature. The effects of FR risk signature on BC immune microenvironment were explored by CIBERSORT algorithm and ssGSEA method. The histological expressions of FR risk genes were presented by HPA database. The biofunctions of SQLE were determined by qPCR, MTT, wound-healing and Transwell assays.

**Results:**

We constructed a novel FR risk signature consisting of eight genes. High FR risk led a poor prognosis and was identified as an independent prognostic factor. Besides, A higher proportion of patients with luminal A type was observed in low-risk group (53%), while a higher proportion of patients with basal type in high-risk group (24%). FR risk score could discriminate the prognostic difference of most clinical subgroups, except for M1 stage, HER2 and basal types. Moreover, its prognostic value was successfully validated in other four cohorts. Through immune analyses, we found that the reduced infiltration levels of CD8+ and NK cells, whereas the enhanced activity of antigen presentation process appeared in high FR risk. Then, FR risk score was found to weakly correlate with the expressions of six immune checkpoints. Through the experiments in vitro, we confirmed that overexpression of SQLE could promote, whereas blocking SQLE could inhibit the proliferative, migrative and invasive abilities of BC cells.

**Conclusions:**

FR risk signature was conducive to BC prognostic assessment. High FR risk level was closely associated with BC immunosuppression, but may not predict ICIs efficacy. Moreover, SQLE was identified as a crucial cancer-promoting gene in BC. Our findings provide new insights into prognostic assessment and molecular mechanism of BC.

**Supplementary Information:**

The online version contains supplementary material available at 10.1186/s12885-021-08892-4.

## Introduction

Breast cancer (BC) is the most common cancer in women worldwide, accounting for 25.4% of all female cancer cases, which places a heavy burden on patients’ health and economy [1]. In United States, the number of new cases of BC in 2020 was about 27,000, and led up to 42,000 cancer-related deaths, contributing to 14.7% of the total cancer-associated mortalities [2]. It is alerting that the incidence of BC has a non-neglected growth in past decade (an average of 0.3% per year). Its age-standardized incidence rate (ASR) increased from 39.2/100,000 to 45.9/100,000 [3, 4]. Although the treatment strategy and approach of BC have been greatly improved, the mortality rate of BC is still up to 1.34 per million in 2019, what’s more, its cancer-related deaths do not decrease [5]. Metastasis is the primary cause of death in BC patients, whereas, about 15% of patients present locally advanced or metastatic symptoms at the time of diagnosis, which brings great challenges for cancer treatment. Therefore, it is extremely urgent and significant to explore the molecular mechanism of BC progression, search for novel therapeutic targets and establish a precise prognostic analytical system.

Ferroptosis is a mode of programmed cell death. Distinct from apoptosis and autophagy, ferroptosis commonly does not cause nuclear condensation and fragmentation, but exhibits mitochondrial abnormalities as the dominant features, such as rupture of the outer membrane, mitochondrial condensation and diminished or vanished of mitochondria crista *etc* [6]. Since the term was first coined by Dixon SJ et al. in 2012, ferroptosis has been proven to be closely related to cancer progression and has great potential to conquer the tumor [7]. Iron ion transport, lipid oxidation and dysfunction of antioxidant pathways act as three crucial links of ferroptosis, which are regulated by ferroptosis-related (FR) genes. It has been found that these regulators are involved in the onset and development of multiple cancers [8]. For example, NCOA4, a gene with the ability to facilitating the release of Fe^2+^ ion through ferritinophagy, is down-regulated in renal cancer tissue, and its deletion leads unfavorable prognosis and immune tolerance [9]. Although recent studies have preliminarily investigated the actions of FR genes in BC [10–13], there are still some issues worthy of further discussion and improvement, such as screening strategy of FR gene set, constructing and assessing process of prognostic model, and functional validations of hub FR genes etc. Herein, we made reasonable improvements on these issues. Meanwhile, we explored the relationships between FR risk score and molecular subtypes of BC for the first time and contrasted the similarities and differences between our work and recent studies.

In the present study, a novel FR risk signature was constructed by using 1109 BC samples from TCGA database. Afterwards, we explored the effects of FR risk signature on prognosis and immune microenvironment of BC, and its prognostic value was also validated in METABRIC, GSE58812, GSE20685 and ICGC-KR cohorts. As for treatment, given that only a small subset of patients can benefit from immune checkpoint inhibitors (ICIs), the potential links between FR risk and the therapy of ICIs were investigated. Furthermore, due to the eager attention attracted from SQLE in oncology field, its pro-oncogenic biofunctions in BC cells were also confirmed. Hence, we believe that our findings will provide valuable insights into the prognostic analysis, therapeutic selection, and molecular mechanism of BC.

## Materials and methods

### Data source

In the present study, the TCGA dataset was used as training cohort, while METABRIC, GSE58812 [14], GSE20685 [15] and ICGC-KR datasets were applied as validation cohorts. In TCGA database, the types of transcriptome and clinical data were set as ‘HTSeq-FPKM’ and ‘Bcr-Xml’, respectively. There were no limits on the pathological type of BC. A total of 1109 BC and 113 normal samples were preliminarily included in the training cohort. Among that, 80 BC samples were excluded due to their too short follow-up period (less than 30 days) or abnormal survival information (survival time is negative value). METABRIC dataset provided transcriptomic and survival information of 1764 BC samples. GSE58812 and GSE20685 datasets consisted of 107 TNBC (triple-negative breast cancer) and 327 BC samples, respectively. ICGC-KR dataset offered another validation cohort containing a total of 50 BC samples. The clinical characteristics of these datasets were shown in Table 1. All gene expression data was processed through log2 transformation.

**Table 1:** The clinical characteristics of TCGA, METABRIC, GSE58812, GSE20685 and ICGC-KR cohorts

	TCGA	METABRIC	GSE58812	GSE20685	ICGC-KR
Sample					
Tumor	1109	1764	107	327	50
Normal	113	148	0	0	50
Survival Status					
Dead	144	1027	29	83	10
Alive	933	737	78	244	40
Age (Median)	58.38	61.41	59.96	47.89	31.81
<60	575	761	64	278	50
≥ 60	502	1003	43	49	0
Grade					
G1	/	154	/	/	/
G2	/	664	/	/	/
G3	/	882	/	/	/
Unknown	/	64	/	/	/
T					
T1/Tis	275	/	/	101	19/3
T2	624	/	/	188	24
T3	136	/	/	26	3
T4	39	/	/	12	1
Unknown	3	/	/	/	/
N					
N0	507	/	/	137	/
N1–3	550	/	/	190	/
Unknown	20	/	/	/	/
M					
M0	897	/	76	244	/
M1	21	/	31	83	/
Unknown	159	/	/	/	/
Clinical Stage					
Stage I	179	436	/	/	13
Stage II	609	745	/	/	28
Stage III	246	111	/	/	6
Stage IV	19	7	/	/	0
Unknown	24	465	/	/	/
Subtype					
Luminal A	497	679	/	/	/
Luminal B	197	461	/	/	/
Basal	171	199	/	/	/
Her2	77	220	/	/	/
Unknown	135	claudin-low = 199 Unknown = 6	/	/	/
OS (Year)	Median = 3.273	Median = 10.428	Median = 6.035	Median = 7.89	Median = 7.558
PFS (Month)	Median = 37.859	/	/	/	/
DFS (Month)	Median = 37.851	/	/	/	/

*OS* overall survival, *PFS* progression free survival, *DFS* disease free survival

### Screening for ferroptosis regulators

**Fig. 1:**
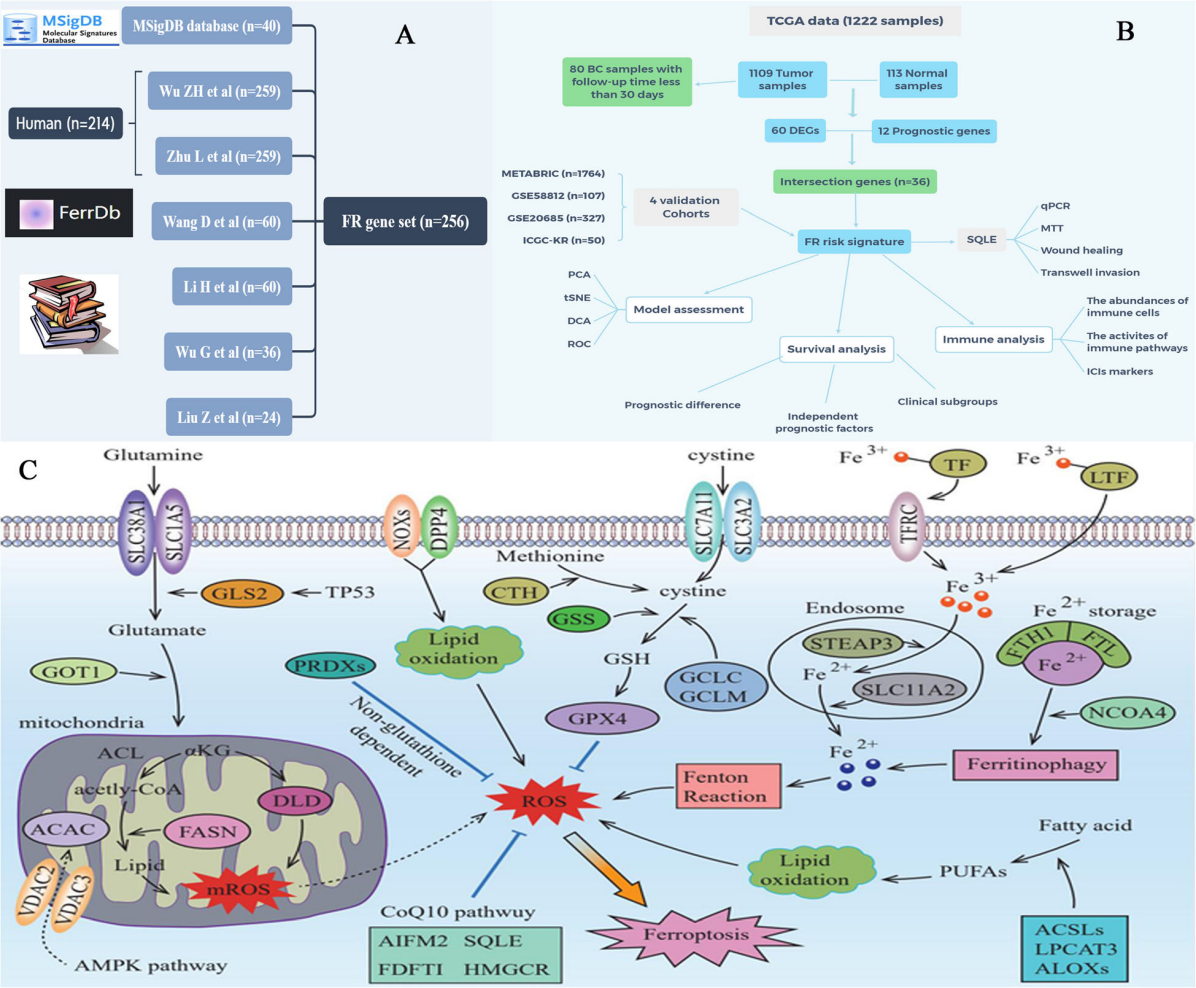
Ferroptosis is a new form of programmed cell death. (A) The establishment of FR gene set. (B) The flow chart of the present study. (C) The regulatory mechanism of ferroptosis. BC, breast cancer; FR, ferroptosis-related; DEGs, differentially expressed genes; PCA, principal component analysis; t-SNE, t-distributed stochastic neighbor embedding; DCA, decision curve analysis; ROC, receiver operating characteristic curve; ICIs, immune checkpoint inhibitors; ROS, reactive oxygen species

In the current study, we established a ferroptosis-related gene (FRG) set based on four sources (Fig. 1A): [1] FerrDb database is the world’s first database of ferroptosis regulators and markers, which provides 108 ferroptosis driver, 69 ferroptosis suppressors, and 111 ferroptosis markers in total (http://www.zhounan.org/ferrdb/) [16]. After removing duplicate genes, the database provides a FR gene set containing 259 genes. Zhu L et al. and Wu Z et al. both adopted this gene set to constructed FR risk signatures [12, 13]. However, FerrDb gene set includes not only human species, but also mice and drosophila species. In the present study, we just selected human ferroptosis regulators (*n* = 214) into our FR gene set, which is strikingly different from previous strategy [12, 13]. Siegel et al. [2] The Molecular Signatures Database (MSigDB) is a collection of annotated gene sets for use with GSEA software [17], which provides a FR set consisting of 40 genes [3]. Several crucial reviews have elaborated the molecular mechanism and regulatory process of ferroptosis [18–22]. Chen et al. [4] Some bioinformatic research focusing on ferroptosis has offered different referencing strategies (Fig. 1A). In a pan-cancer study, Liu Z et al identified 24 critical genes in ferroptosis-process [23]. Wang D et al. and Li H et al. applied a completely same FR gene set consisting of 60 regulators to explore the roles of FR genes in BC [10, 11]. Wu G et al. constructed a FR gene set consisting of 36 regulators in renal cancer research [24]. Finally, in light of these research, we constructed an improved FRG set containing 256 genes which almost covered all genomes above. The establishment process of our FRG set, and the member information of all FRG sets above were presented in Fig. 1A and Supplementary Table 1.

### The construction of ferroptosis-related risk signature

The expressive difference of FRGs between BC tumor and normal samples was compared using ‘Limma’ package in R software (Ver 3.6.3). When absolute value of Log_2_FC ≥ 1.0 and adjusted *p* value < 0.05 were satisfied simultaneously, the genes were regarded as the differential expressed genes (DEGs). Next, we screened out the genes affecting BC prognosis by performing cox univariate regression analysis, namely prognostic FRGs. Venn diagram was employed to identify the intersection between FR DEGs and prognostic FRGs. Finally, based on the Lasso regression analysis, these intersection genes were used to construct a novel FR risk signature via the “glmnet” R package.

### Model assessment

The predictive accuracy of FR risk signature was evaluated through the receiver operating characteristic curve (ROC). Principal component analysis (PCA) and t-distributed stochastic neighbor embedding (t-SNE) can show the stratifying performance of FR prognostic model. Furthermore, when we introduced FR risk score into BC prognostic analysis, its clinical benefit was quantified through decision curve analysis (DCA).

### Survival analyses

Survival analysis was based on the Kaplan–Meier method and was conducted through ‘survminer’ and ‘survival’ R packages. The optimal cutoff value of risk score was used as grouping criteria through ‘Cutoff Finder’ online tool (https://molpathoheidelberg.shinyapps.io/CutoffFinder_v1/) [25]. Cox univariate and multivariate analyses were successively conducted to confirm that whether FR risk score was an independent prognostic factor of BC. To determine the applicable domain of the novel FR prognostic model, we compared the survival difference between high- and low-risk groups in the same clinical subgroup. Moreover, we established a nomogram combining BC clinicopathological features and FR risk signature to predict the overall survival rate (OSR) of individual at 1,3,5 year. Calibration plot was implemented to test whether the nomogram can well compare with an ideal prognostic model. The risk plots were displayed by using ‘pheatmap’ R package. Besides, the distributions of genetic expressions, survival outcomes and clinicopathological features in different risk groups were also presented via ‘pheatmap’ R package.

### Immune analyses

We evaluated the effects of high FR risk on BC immune microenvironment in three aspects. First, the immune abundances of 22 lymphocyte subtypes in each BC sample were calculated based on the CIBERSORT algorithm [26]. The difference in immune cell infiltration between high and low risk groups was tested via the ‘Limma’ package in R software. Second, we determined the correlations between FR risk score and the infiltration levels of six crucial cancer-related immune cells based on Spearman method. Third, the active scores of 13 immune-related pathways were calculated based on single-sample gene set enrichment analysis (ssGSEA) [27]. Then, we evaluated the alteration in pathway activity brought by high FR risk through ‘Limma’ and ‘ggradar’ R packages.

To speculate the roles of FR risk signature in predicting the efficacy of ICIs, we investigated the relationships between FR risk level and six pivotal immune checkpoints (PD-L1, CTLA4, BTLA, LAG3, HAVCR2 and TIGIT). Their expressive correlations with FR risk score, and the expressive distributions in different FR risk levels were both confirmed via ‘ggplot2’ and ‘ggpubr’ R packages.

### HPA database

The human protein atlas (HPA) database can achieve spatial localization of proteins down in the single-cell level and provide histological expression information of genes (https://www.proteinatlas.org/) [28]. The protein expression levels of FR risk genes in BC and normal tissues were visualized via the immunohistochemistry images in HPA database [29].

### Cell culture and transfection

One normal human breast cell line (MCF-10A) and two breast cancer cell lines (MCF7 and MDA-MB-231) were purchased from Procell Life Science and Technology Company (Wuhan, China). MCF-10A cells were cultured in Dulbecco’s Modified Eagle’s Medium (DMEM) containing 10% fetal bovine serum (FBS) and 1% Penicillin/Streptomycin (P/S). MCF7 cells were cultured in Minimum Essential Medium (MEM) containing 10%FBS and 1% P/S. MDA-MB-231 cells were cultured in Leibovitz’s L-15 medium containing 10%FBS and 1% P/S. All cells were incubated at 37 °C with 5% CO^2^ and 95% humidity.

We applied siRNAs to inhibit the mRNA expression of SQLE. Specific interfering RNA fragment target SQLE (si-SQLE) was designed and synthesized by GenePharma Biotechnology (Shanghai, China). Lipofectamine™ 2000 was employed to perform transfection (Thermo Fisher Scientific, Waltham, MA, USA). The plasmids pcDNA-SQLE were designed and purchased from GeneChem (Shanghai, China) for SQLE overexpression.

### Real-time quantitative PCR (RT-qPCR)

Total RNA was extracted through TRIzol reagent (Thermo Fisher Scientific, Waltham, MA, USA). RT (Reverse Transcription) reagent Kit (Takara, Japan) was applied to synthesized cDNA. Target sequence was amplificated via SYBR-Green reagent (Takara, Japan) and PCR reaction was detected on the ABI PRISM 7900 System (Thermo Fisher Scientific, CA, USA). Expression levels were normalized to GAPDH and the relative mRNA levels were calculated based on 2^−ΔΔCt^ method. Primer list was presented in Supplementary Table 2.

### MTT assay

Transfected cells were seeded in 96-well culture plates with the concentration of 5 × 10^3^/well and were cultivated for 24, 48, 72 and 96 h. At each time point, MTT Reagent (Solarbio Life Science co, Beijing, China) was added in plates and treated cells for 4 h, at 37 °C. Then, the medium was discarded and 150 μL DMSO was added. The absorbance was measured by a microplate reader (ThermoFisher, Waltham, MA, USA) at 490 nm.

### Wound-healing assay

Transfected cells (1 × 10^4^/well) were seeded in 6-well plates and 2 ml medium was added in each well. After overnight incubation, the cells were stably adherent on the plates. Discarding medium and creating a linear wound via a sterile 200 μL pipette tip. Floating cells and cellular debris were removed by twice PBS washing and serum-free medium was added. After 24 h incubation, cell migration process was observed under a microscope. Cell migrative ability was quantified by calculating the wound width rate. The wound width rate = (the scratch width at 0 h *minus* that at 24 h *divided by* initial width) × 100%.

### Transwell invasion assay

Transwell chambers (Corning, NY, USA) were placed in 24-well plates. One hundred μL DMEM/MEM-diluted Matrigel (Corning, NY, USA) was added in each chamber and incubated overnight for gelling. Transfected cells (5 × 10 ^4^/ well) were seeded in upper chamber with serum-free medium. The lower chamber contained 500 μL DMEM/MEM with 10% FBS. After incubation for 36 h at 37 °C, medium in upper chamber was removed and Transwell chambers were washed twice by PBS. The invasive cells were fixed by paraformaldehyde for 20 min and stained by 0.1% crystal violet for 20 min. Then, remaining non-invasive cells were wiped out by a cotton swab. The invasive cells per three random fields of view were counted under a microscope at 100 × magnification.

### Statistical analysis

All statistical analyses were conducted using R software (Version 3.6.2) and GraphPad Prism (Version 8.01). Student’s t-test or Chi-square test were used to determine the differences among variables. *P*-value < 0.05 was regarded as statistically significant. All in vitro assays were repeated in triplicate.

## Results

Using 1222 samples from TCGA database, we constructed a novel FR risk signature (including SQLE, ALOX15B, ANO6, TP63, JUN, PLIN4, ACSL1 and CHAC1). Its prognostic value, effects on immune microenvironment and associations with immune checkpoint markers were all explored. The predictive ability of FR prognostic model was also validated in four extra cohorts. Given that the great potential of SQLE in cancer therapy and the close attention to SQLE from oncology field, its expressions and biofunctions were investigated through a series of experiments in vitro. The flowchart of the current study was shown in Fig. 1B.

### Ferroptosis is a distinct mode of programmed cell death

In 2003, Dolma, S et al. firstly found that erastin can induce a nonapoptotic cell death process [30]. However, until 2013, Dixon, S et al. just formally designated the new type of cell death as ‘Ferroptosis’ [18]. Ferroptosis involves three crucial processes, including iron ion transport, lipid oxidation and dysfunction of antioxidant pathways. First, lipid oxidation of cell membranes promotes the accumulation of reactive oxygen species (ROS), which drives ferroptotic cell death. However, not all kinds of fatty acids (FAs) can be oxidized in ferroptosis process, only polyunsaturated fatty acids (PUFAs) in phospholipids are susceptible to oxidative damage [31]. ACSL and ALOX families both participated in the synthesis of lipid precursor required for ferroptosis [19]. Besides, LPCAT3 is also proven to make prominent contributions through lipid remodeling in above process [32]. Second, Fe^2+^ ion is responsible for receiving electron needed for lipid oxidation, hence, iron transport can markedly regulate ferroptosis process. Fe^3+^ binds to transferrin (TF) in the serum and can be recognized by TFRC (transferrin receptors) in the cell membrane. On one hand, the intracellular Fe^3+^ locating in endosome reduces to Fe^2+^ via the catalyzing of STEAP3 [33], and releases it into the cytosol through SLC11A2 [34]. On the other hand, ferritin, namely the iron-storage protein, also can release Fe^2+^ through NCOA4-mediate ferritinophagy [35]. Third, GPX4 can serve as a reductase to antagonize lipid oxidation, whose expression and activity are controlled by GSH [20]. While the precursor of GSH originates from cystine, which is transported by SLC7A11 and SLC3A2 [22]. Therefore, GSH depletion and GPX4 inactivation facilitate ferroptosis. The main mechanism of ferroptosis was exhibited in Fig. 1C.

### A novel ferroptosis-related risk signature is constructed

**Fig. 2:**
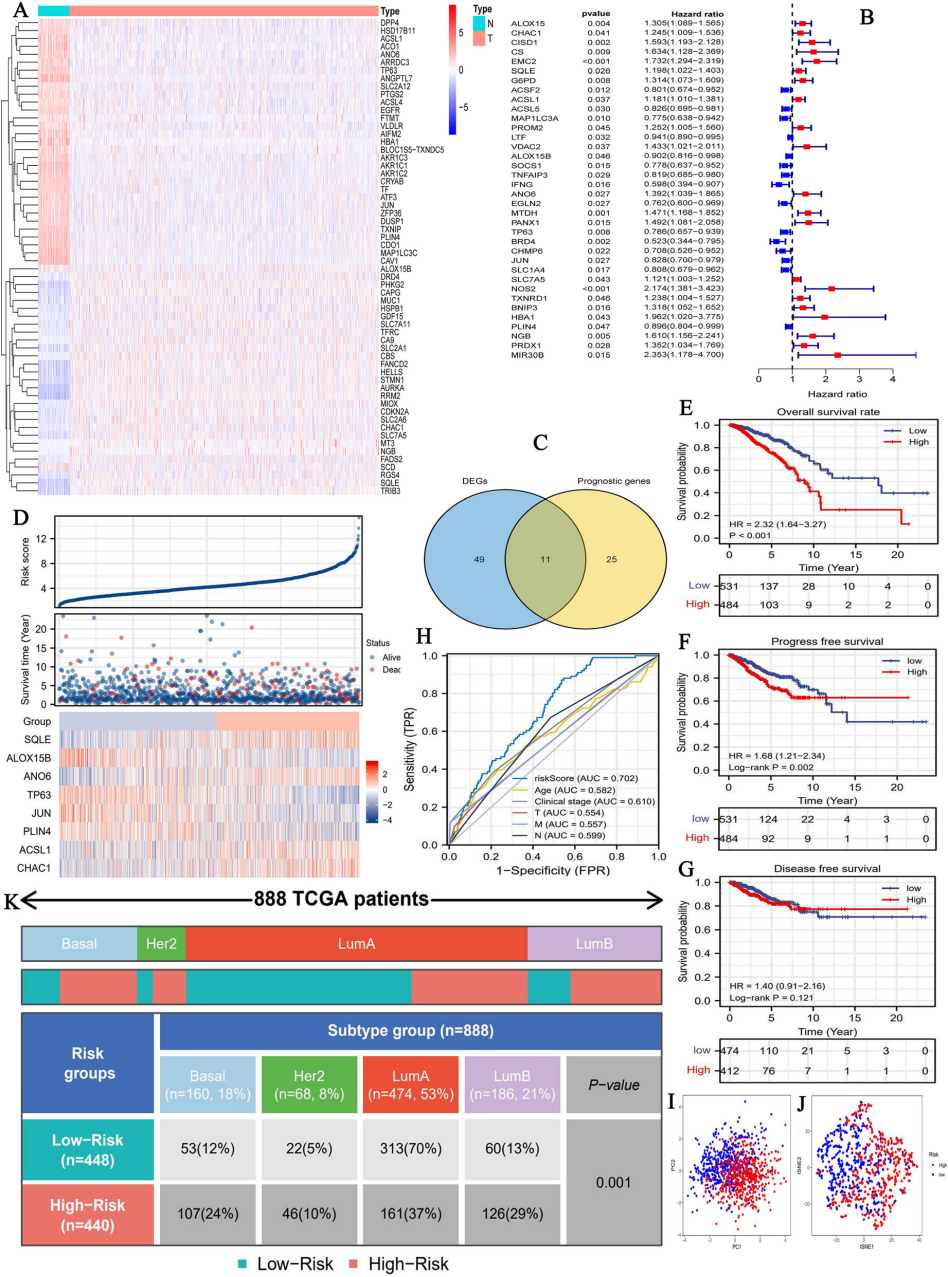
The construction of FR risk signature. (A) The heatmap of FR DEGs. (B) Identification of prognostic FR genes via cox univariate analysis. (C) The intersection genes between FR DEGs and FR prognostic genes. (D) The risk plots of FR risk signature. (E) The overall survival difference between high- and low-FR risks. (F) The progression-free survival difference. (G) The disease-free survival difference. (H) The ROC of FR risk signature. (I) The PCA of FR risk signature. (J) The t-SNE analysis of FR risk signature. (K) The distributions of molecular subtypes in different risk groups. FR, ferroptosis-related; DEGs, differential expressed genes; PCA, principal component analysis; t-SNE, t-distributed stochastic neighbor embedding; ROC, receiver operating characteristic curve; Lum A, luminal A; LumB, luminal B

Comparing to normal samples, 60 of 256 FRGs (23.4%) were differentially expressed in BC samples (Fig. 2A). Among 22 DEGs, 27 FRGs were upregulated, and others were downregulated. Through cox univariate analysis, 36 of 256 FRGs (14.1%) were found to be closely related to BC prognosis (Fig. 2B). Fifteen FRGs favored patients’ prognosis. In contrast, other 21 FRGs were unfavorable for patient survival. Then, we identified 11 intersection genes between FR DEGs and prognostic FRGs, containing SQLE, CHAC1, ACSL1, ALOX15B, ANO6, TP63, JUN, SLC7A5, PLIN4, NGB and HBA1(Fig. 2C). Of note, NGB (*n* = 333) and HBA1(*n* = 388) expressions in more than one-third of BC samples presented zero FPKM values. To preventing their interference with establishing FR prognostic model, they were not filtered by LASSO regression analysis.

Finally, a novel ferroptosis-related risk signature was constructed based on Lasso regression analysis (Supplementary Fig. 1), as follows: FR risk score = 0.0583 × (relative expression of SQLE) + (− 0.0583) × (relative expression of ALOX15B) + 0.373 × (relative expression of ANO6) + (− 0.0984) × (relative expression of TP63) + (− 0.0458) × (relative expression of JUN) + (− 0.0064) × (relative expression of PLIN4) + 0.0411 × (relative expression of ACSL1) + (0.0906) × (relative expression of CHAC1).

### Ferroptosis-related risk signature contributes to prognostic assessment of breast cancer patients

Using FR risk signature, the risk score of each BC sample in TCGA cohort was calculated (Fig. 2D). 1015 BC patients were stratified into high- (*n* = 484) and low-risk groups (*n* = 531) according to the optimal cutoff value of risk score (4.292). High risk level led a poor survival outcome with a 5-year OSR of 78.1%, whereas that in low-risk group was 89.6% (Fig. 2E). Moreover, there was a significant survival advantage on PFS (progression-free survival) in low-risk group compared to high-risk group (Fig. 2F). Nevertheless, this trend was not observed in DFS (disease-free survival) (Fig. 2G). Next, we evaluated the predictive accuracy of the novel prognostic model. An area under the ROC (AUC) of risk score was 0.702, which suggested a better prognostic analytical performance of risk score than that of other traditional clinical parameters (Fig. 2H). PCA and t-SNE analyses indicated that patients with different risk groups possessed notably different prognostic clustering (Fig. 2IJ). It is worth mentioning that there was a markedly difference in the distribution of BC molecular subtypes between high- and low-risk groups (Fig. 2K). A higher proportion of patients with luminal A type was observed in low-risk group (53%), while a higher proportion of patients with basal type in high-risk group (24%) (Fig. 2K).

**Fig. 3:**
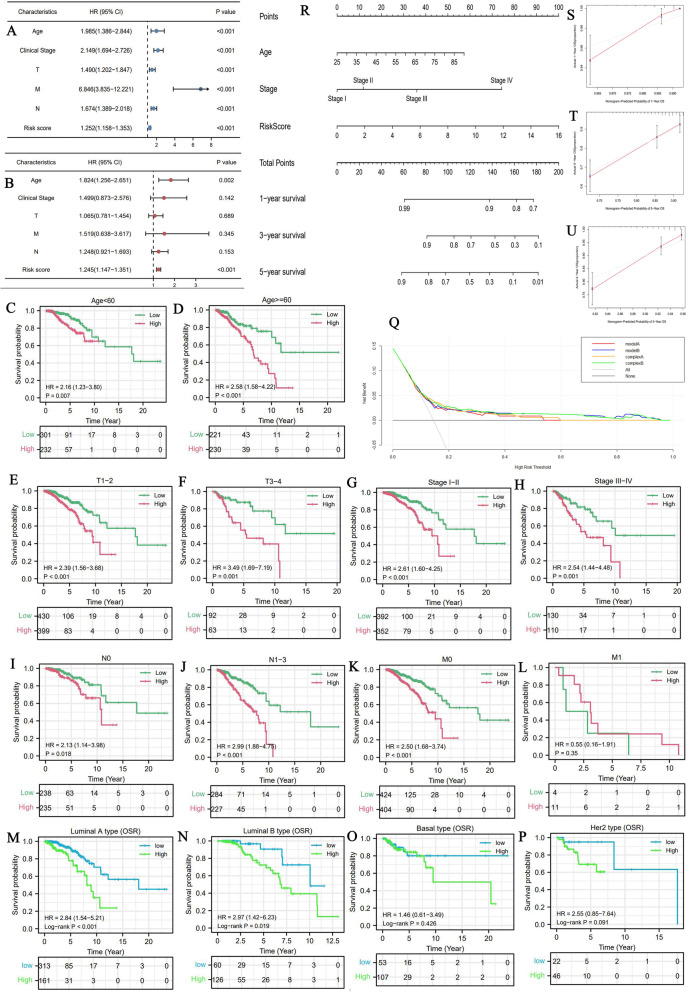
FR risk signature provides an important supplement to prognostic analysis of BC. (A) The result of cox univariate analysis in TCGA cohort. (B) The result of cox multivariate analysis in TCGA cohort. (C-L) FR risk signature can distinguish the prognostic differences of BC patients with most clinical subgroups. (M-P) The overall survival differences of patients with four molecular subtypes between high- and low-FR risk groups. (Q) The DCA of FR risk signature. Different curves represent four kinds of BC prognostic models based on multivariate logistic regression analysis. Model A represents the prognostic model composed of age and TNM stages. Model B represents the prognostic model composed age and clinical stage. Complex A represents the improved model A that added FR risk score. Complex B represents the improved model B that added FR risk score. (R) The nomogram composed of age, clinical stage, and FR risk score. (S-U) The calibration curves. FR, ferroptosis-related; BC, breast cancer; DCA, decision curve analysis

Going one step further, risk score and age were both identified as independent prognostic factors of BC (HR_riskscore_ = 1.245, *P*<0.001; HR_age_ = 1.824, *P* = 0.002) (Fig. 3AB). Meanwhile, FR risk signature was proven to have a good applicable range. It could discriminate the prognostic differences of BC patients with most clinical subgroups (Fig. 3C-K), except for patients with M1 stage (Fig. 3L). DCA analyses revealed that introducing FR risk into traditional prognostic model (model A or B) could slightly increase clinical benefit when making clinical-decision (Fig. 3Q). As for different molecular subtypes, the FR prognostic model was able to effectively work in luminal A and B types, but not in basal and HER2 types (Fig. 3MP).

Finally, we established a nomogram to predict the 1,3,5-year OSR of BC patients (Fig. 3R). For example, a 70-year old (40 points) BC patient who was diagnosed as clinical stage II (12.5 points) and tested with a risk score of 8 (50 points) will get a total point of 102.5, whose 5-year OSR is estimated less than 70%. Calibration plots revealed that predicted OSRs were similar to the actual survival rate (Fig. 3S-U). In a word, all these results reiterated that FR risk signature could contribute to prognostic assessment of BC patients.

### The prognostic value of risk signature is successfully verified in multiple validation cohorts

**Fig. 4:**
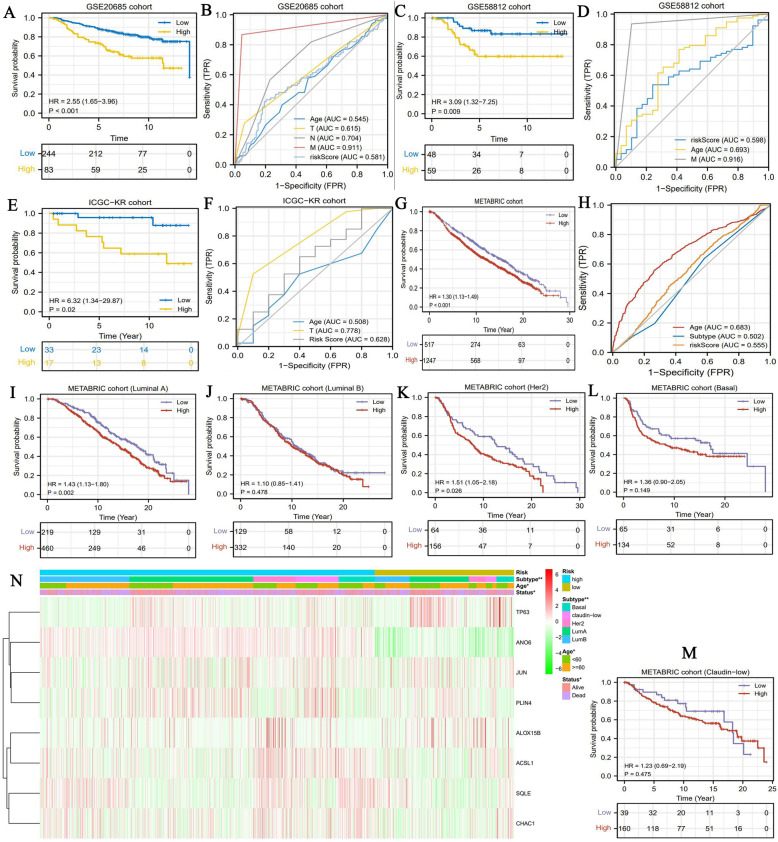
The prognostic value of FR risk signature is validated in multiple cohorts. (A) Survival difference in GSE20685 cohort. (B) ROC curve in GSE20685 cohort. (C) Survival difference in GSE58812 cohort. (D) ROC curve in GSE58812 cohort. (E) Survival difference in IGCG-KR cohort. (F) ROC curve in ICGC-KR cohort. (G) Survival difference in METABRIC cohort. (H) ROC curve in METABRIC cohort. (I-M) The survival differences of patients with five molecular subtypes in METABRIC cohort. (N) The heatmap for FR gene expressions and clinical features in METABRIC cohort. FR, ferroptosis-related; ROC, receiver operating characteristic curve; * means *P*<0.05; ** means *P*<0.01

In order to validate the prognostic value of FR risk signature, we have conducted the prognostic analyses in four distinct datasets which contained a total of 2248 samples. In GSE20685 cohort, high FR risk offered an unfavorable survival outcome (Fig. 4A). Nonetheless, FR risk score did not have a preponderance on prognosis prediction compared to TNM staging system (AUC = 0.581) (Fig. 4B). In GSE58812 and ICGC-KR cohorts, although high FR risk both resulted in poor prognosis (Fig. 4CE), their predictive accuracy were still not excellent (AUC = 0.598 and 0.628) (Fig. 4DF).

METABRIC dataset served as a core validation cohort for its sufficient sample information (*n* = 1764). As previously found in other cohorts, high FR risk was an adverse prognostic factor (*P <* 0.001, HR = 1.30) (Fig. 4G). Regarding prediction accuracy, FR risk signature possessed an AUC of 0.555, which was lower than that in TCGA cohort (AUC = 0.702). Moreover, FR risk level was also closely associated with molecular subtypes of BC. The FR model could distinguish the prognostic differences of patients with luminal A and HER2 types, whereas failed to work in patients with luminal B, Basal and claudin-low types (Fig. 4I-M). Basal type accounted for a higher proportion in high-risk group than that in low-risk group (Fig. 4N). Altogether, the prognostic value of FR risk signature was successfully verified in multiple validation cohorts, but its predicting performance decreased.

### High ferroptosis-related risk is unfavorable for antitumor immune response

**Fig. 5:**
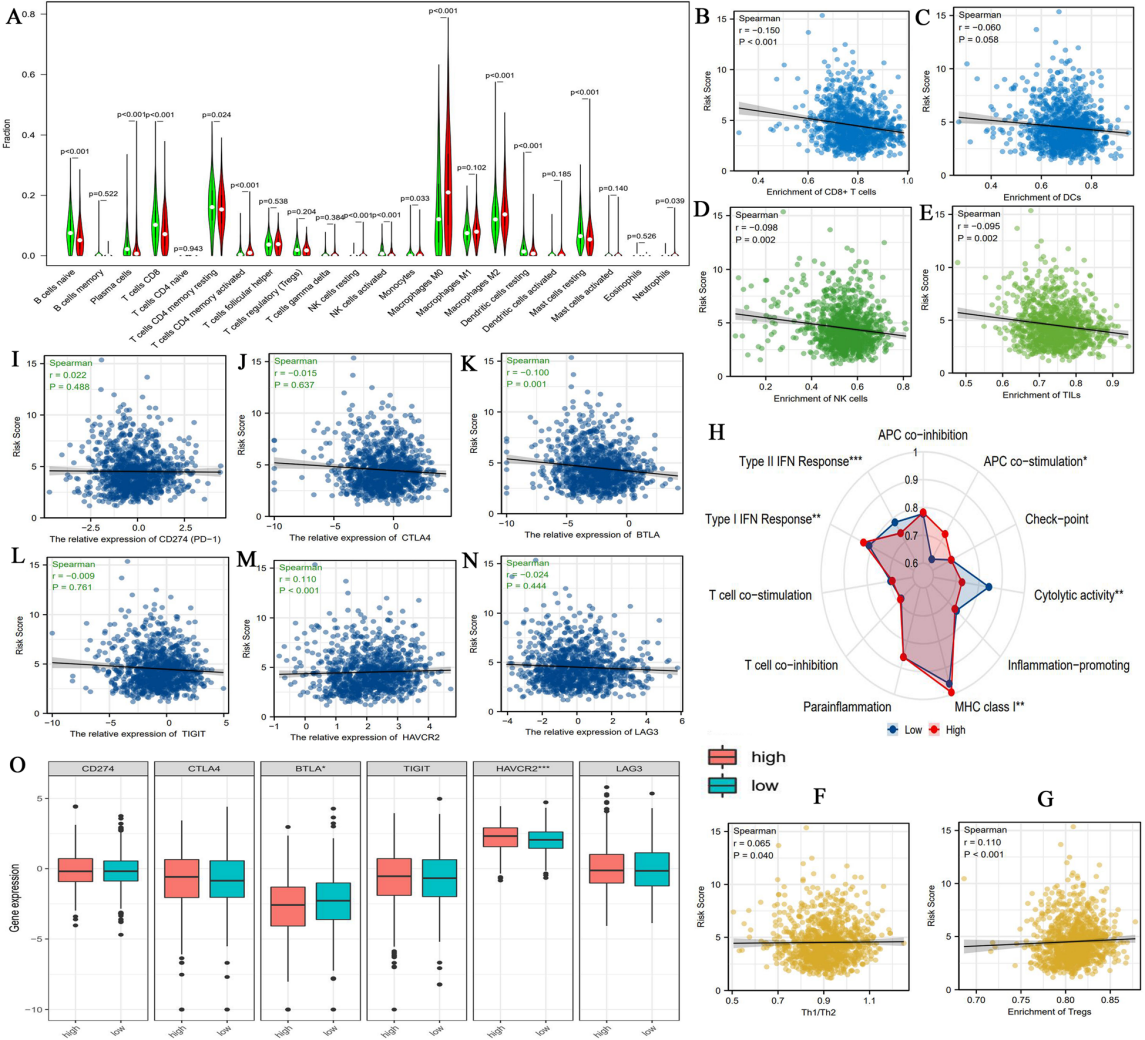
The effects of FR risk levels on TIM and the expressions of immune checkpoints. (A) The differences in infiltrating levels of 22 immune cells between high- and low-risk groups. High-risk group is red and low-risk group is green. (B-G) The correlations between FR risk score and the enrichments of six core immune cells. (H) The differences in activities of 11 immune signaling pathways between high- and low-risk groups. (I-N) The expressive correlations between FR risk score and six immune checkpoints. (H) The expressive differences of six immune checkpoints between high- and low-risk groups. FR, ferroptosis-related; TIM, tumor immune microenvironment; APC, antigen presentation cell; IFN, interferon; MHC, major histocompatibility complex; PD-1 (CD274), programmed cell death 1; * means *P*<0.05; ** means *P*<0.01; *** means *P*<0.001

Further, we evaluated the impact of FR risk level on tumor immune microenvironment (TIM) based on the CIBERSORT algorithm. The immune abundances of 22 leukocyte subtypes in each BC sample were exhibited in Supplementary Fig. 2. The infiltration levels of B naive cells (*P*<0.001), plasma cells (*P*<0.001), T cells CD8 (*P*<0.001), T cells CD4 memory resting (*P* = 0.024), NK cells activated (*P*<0.001), dendritic cells (dendritic cells) resting (*P*<0.001) and Mast cells resting (*P*<0.001) in high risk group were significantly higher than that in low risk group. On the contrary, the high risk level was closely associated with the higher immune abundances of T cells CD4 memory activated (*P*<0.001), NK cells resting (*P*<0.001), Monocytes (*P* = 0.033), Macrophages M0 (*P*<0.001), Macrophages M2 (*P*<0.001) and Neutrophils (*P* = 0.039). In addition, the enrichments of CD8+ T cells, DCs, NK cells and TILs (tumor-infiltrating lymphocytes) were all negatively correlated with FR risk score (Fig. 5B-E). Conversely, the ratio of Th1/Th2 cells and the enrichments of Tregs (T cells regulatory) were positively correlated with FR risk score (Fig. 5FG). As acknowledged, the changes in infiltration levels of immune cells will lead a complex alteration of TIM. Referring to some immune related studies [36–47], we found that the changes in most immune cellular components were detrimental to antitumor process, whereas were conducive to antigen-presenting process and adaptive antitumor immune response (Table 2).

**Table 2:** The effect of alteration of TIM on antitumor immunity

Immune cell	Variation trend in high FR risk	Basic immune function	Final effect on anticancer process
T cells CD8	Decreasing	CD8+ T cells are the main effector cells responsible for killing tumour cells and virally infected cells.	Unfavorable
T cells CD4 memory	Activated Type Increased	Memory CD4 T cells play a crucial role in adaptive immune response.	Beneficial
	Resting Type Decreasing		
NK cells	Activated Type Decreasing	NK cells can provide host defense against tumour through their potent cytolytic function.	Unfavorable
	Resting Type Increased		
Macrophages M2	Increased	M2 cells can facilitate tumor cells proliferation and repair.	Unfavorable
Dendritic cells	Activated Type No change	DCs specialize antigen-presenting process and contribute to adaptive immune response, but may induce immune tolerance.	Uncertain
	Resting Type Decreasing		
Mast cells	Activated Type No change	Mast cells possess pro-tumor or anti-tumor bi-directional abilities via secreting different factors.	Uncertain
	Resting Type Decreasing		
Plasma cells	Decreasing	Plasma cells commonly serve a positive role in antitumor immunity.	Unfavorable
TILs	Negative Correlation	TILs play a specific killing effect on tumors.	Unfavorable
Tregs	Positive Correlation	Tregs play an immune suppressive role through expressing the transcription factor FoxP3.	Unfavorable
Th1/Th2	Positive Correlation	Th1/Th2 balance toward Th1 is beneficial for antitumor immune process.	Beneficial

*TIM* tumor immune microenvironment, *NK cells* natural killer cells, *Tregs* T cells regulatory, *DCs* Dendritic cells, *TILs* tumour-infiltrating lymphocytes

Meanwhile, FR risk level could exert an observable effect on the activities of immune related pathways. As shown in Fig. 5H, cytolytic activity and Type-II IFN response were both suppressed in high FR risk level. Reciprocally, APC co-stimulation, MHC class I, and Type-I IFN response were all facilitated in high FR risk level. These findings reiterated that high FR risk level heralded the weakened antitumor cellular immune but enhanced antigen-presenting process.

Although there is no definitive agreement on biomarkers for predicting ICIs efficacy, patients with PD-1 overexpression commonly present a good therapeutic response to ICIs treatment [48]. Therefore, we investigated the correlations between six crucial immune checkpoints (ICs) and FR risk level. The results revealed that FR risk score was weakly correlated with the expressions of BTLA (*R* = -0.01) and HAVCR2 (*R* = 0.110), while it was not associated with other ICs (Fig. 5I-N). Similarly, BTLA expression was significantly lower in high-FR-risk group than that in low risk group (Fig. 5H). An inverse situation was observed in HAVCR2. Combined with the results above, we speculated that FR level may not serve as a predictive biomarker of ICIs efficacy.

### Some ferroptosis-related risk genes differentially express in histological level

**Fig. 6:**
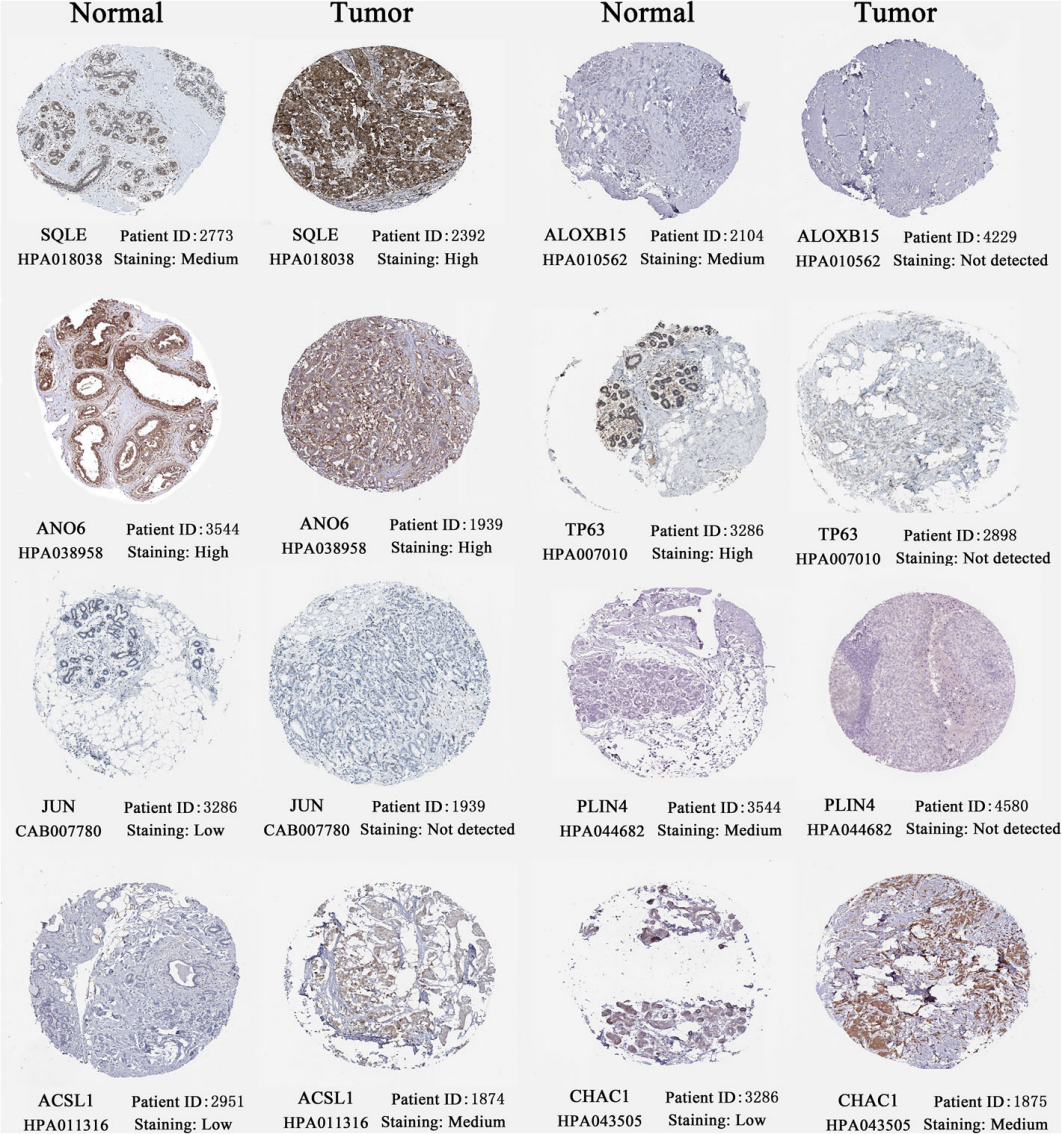
The histological expressions of FR risk genes. The top of the figure indicates the category of tissue specimen. The name of FRG, the antibody type, patient ID, and staining degree are all shown at the bottom of each image. FR, ferroptosis-related; FRG, ferroptosis-related gene

The histological expressions of FR risk genes were displayed in Fig. 6. ALOXB15, TP63 and PLIN4 were obviously downregulated in tumor samples compared to normal ones. JUN presented low-expression in both normal and tumor tissues, whereas ANO6 presented high-expression. Reciprocally, SQLE, ACSL1 and CHAC1 were significantly upregulated in BC tissues compared to normal counterparts.

### SQLE can promote the proliferation, migration and invasion of breast cancer cells

Among five FR risk genes, SQLE, a key rate-limiting enzyme in cholesterol biosynthesis, attracts our attention. In recent years, cholesterol metabolism is proven to play an important role in oncogenic process, ferroptosis, and tumor microenvironment [49]. And its regulatory gene, SQLE, has recently emerged as a promising approach against cancer [50]. Therefore, we further investigated its biofunctions in BC.

**Fig. 7:**
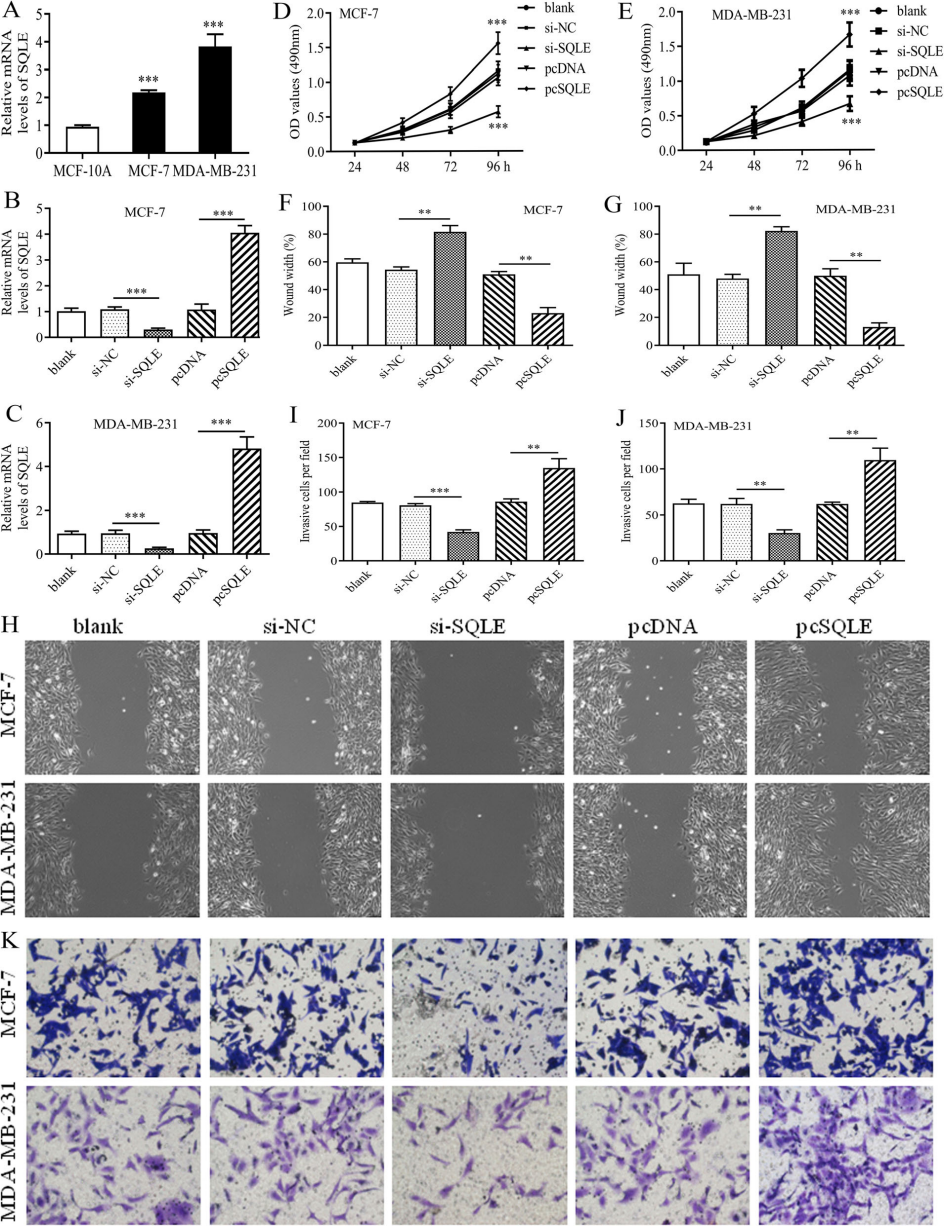
SQLE exerts pro-oncogenic abilities in BC cells. (A) The mRNA expression levels of SQLE in different cells. (B-C) The tests of transfection efficiency in MCF-7 and MDA-MB-231 BC cells. (D-E) The proliferative abilities of MCF-7 and MDA-MB-231 BC cells were assessed through MTT assays. (F-G) The quantitative results of wound-healing assays in 2 BC cells. (H) The images of wound-healing assays. (I-J) The quantitative results of transwell assays in 2 BC cells. (H) The images of transwell assays

As previously found in public databases, SQLE was significantly upregulated in BC cells (MCF7 and MDA-MB-231) compared with normal breast cell line (MCF-10A) (Fig. 7A). Next, si-SQLE and pc-SQLE were shown to effectively alter the expression of SQLE in MCF7 and MDA-MB-231 cell lines (Fig. 7 BC). MTT assays revealed that SQLE overexpression could promote, whereas blocking SQLE could inhibit the proliferation of BC cells (Fig. 7DE). Moreover, SQLE was observed to significantly facilitate the migrative ability of BC cells through wound- healing assays (Fig. 7FGH). As for cell invasion, SQLE overexpression has a stimulative effect on MCF7 and MDA-MB-231 cells, conversely, silencing SQLE has an inhibitory effect (Fig. 7IJK).

### The comparison between four related research and our study

Recently, there are four research have successively probed into the roles of FRGs in prognosis, immune microenvironment of BC. Therefore, we compared these studies with ours, and some improvements in study design and data processing could be noted (Table 3). First, different FR gene sets. The establishment of gene set is the foundation of constructing prognostic model. Li H et al. [10] and Wang D et al. [11] applied a completely same FR gene set (*n* = 60) that may originate from a hepatocellular carcinoma study [51]. Obviously, this gene set could not comprehensively embody the landscape of ferroptosis regulation. FerrDb, the first database of ferroptosis regulators and markers, efficiently compensates for the deficiency. Zhu L et al. [13] and Wu Z et al. [12] both employed FerrDb database to construct a FR gene set containing 259 genes. Nonetheless, this gene set not only contains the ferroptosis regulatory genes derived from human species, but also those derived from mice and drosophila species. In view of these, we constructed a new and improved FR gene set in reference to FerrDb database, MSigDB database and previous research. Second, validation cohorts with maximum sample size. As shown in Table 3, we used four validation cohorts from three sources, including METABRIC, ICGC and GEO databases, to test the prognostic value of FR model. Undoubtedly, it expanded the validation range to increase the credibility of FR model. Third, support from experiment in vitro. Zhu L et al. [13] have detected the changes in mRNA expressions of 11 FR risk genes under Erastin, a ferroptosis inducer, exposure, but not further verified their biofunctions in BC cells. In the present study, we confirmed the oncogenic effects of SQLE in BC progression, whereas other three studies did not perform any experimental validation. Fourth, compared with other existing research, we investigated the links between FR risk level and molecular subtypes of BC. Patients with high risk level possessed a greater propensity for categorizing into basal type, a treatment-resistant subtype (Fig. 2K). Meanwhile, the FR model had a preponderance of prognosis analytical ability in luminal A type over that in basal type (Fig. 3M-P). Besides, we adopted a more reasonable approach of data processing, such as grouping criterion, standardization of expression matrix, and deletion of abnormal samples (Table 3).

**Table 3:** The comparison between four related research and our study

Item/Study	Li H et al	Wang D et al	Zhu L et al	Wu Z et al	Ours
PMID	33,672,990	34,059,009	34,222,241	33,947,836	NA
Publishing date	2021.02	2021.06	2021.07	2021.05	NA
FR gene set	60	60	259	259	256
Species of FRGs	Human	Human	Human/Mice /Drosophila	Human/Mice /Drosophila	Human
Risk signature	8	9	11	15	8
Grouping criterion	Median	Median	Median	Median	Optimal cutoff value
ROC (1Y/2Y/3Y)	None	0.618/0.653/0.663	0.7/0.749/0.72	0.719	0.702
Validation cohorts	METABRIC (*n* = 1904)	GSE42568 ICGC (*n* = 154)	GSE20685 GSE20711 GSE42568 (*n* = 519)	None	METABRIC GSE20685 GSE58812 ICGC (*n* = 2248)
Experiments in vitro	None	None	Alterations of FRGs expressions under Erastin inducing	None	SQLE biofunctions
Data screening	None	Exclude samples follow up with 0 day	None	None	Exclude samples follow up less than 30 days

*FR* ferroptosis-related, *FRGs* ferroptosis-related genes, *NA* not applicable, *1Y/2Y/3Y* time-dependent ROC, AUC at 1-year/2-year/3-year

It is noteworthy that the predictive performance of our model was not optimal. It has a slight disadvantage over Zhu L’s and Wu Z’s models (0.702 vs 0.749/0.719). However, the gene number of our model is smaller than that of Zhu L’s and Wu Z’s models (8 vs 11/15) (Supplementary Table 3). Taking this fact into account, the calculation of FR risk score based on our model is simpler than that based on their models. Collectively, our findings provide some new insights into FR research.

## Discussion

Breast cancer is most common tumor in women. Although the therapeutic means has been obviously improved, the number of cancer-related death is not yet significantly decreased and there is still much room for improving patients’ prognosis, especially for metastatic cases. Therefore, it is still worthy of exploring the molecular mechanism of BC progression and establishing reasonable prognostic assessment system. Ferroptosis is an iron-dependent, lipid oxidation-driven pattern of cell death, which offers a new landscape for treating cancer. Unfortunately, its roles in BC are not yet fully clarified. Hence, the original intention of this study was to preliminarily reveal the effects of FRGs in prognosis, progression and immune microenvironment of BC.

It is defective to performing prognostic analysis of BC patients solely depend on TNM staging system. In fact, the eighth edition of the AJCC TNM system has already integrated some crucial biomarkers with anatomic definitions, the added ones including estrogen receptor (ER), progesterone receptor (PR) and HER2 [52]. When we obtain the information of these biomarkers postoperatively and proceed pathologic staging, it will change the initial staging of 40% of patients, which undoubtedly increases the accuracy of prognostic assessment [52]. In the current study, the novel FR risk signature we constructed was not only identified as an independent prognostic factor of BC, but also could distinguish the survival differences of patients in most clinical subgroups (Fig. 3). DCA analysis also indicated that the risk signature could increase the net benefit when making clinical decision (Fig. 3Q). Furthermore, the prognostic value of FR risk signature was also validated in a TNBC cohort (GSE58812). TNBC is a special subtype of BC with highly aggressive and metastatic abilities, accounting for 15 to 20% of all BC patients. Due to its high heterogeneity, selecting the effective biomarkers for predicting survival outcomes is not easy, meanwhile, several promising biomarkers have not yet been validated through clinical trials [53]. Hence, our FR risk signature may provide some new insights to the issue. Besides, we developed a nomogram so that we could straightforwardly and conveniently predict the 1,3,5-year OSR of BC patients, which has a certain clinical application. Altogether, the novel FR risk signature is an important supplement for the prognostic evaluation of BC.

Intriguingly, we found a closely relationships between FR risk score and BC molecular subtypes. Different molecular subtypes herald distinct prognosis, more importantly, guide strikingly different therapeutic strategies. For metastatic BC patients, median survival of luminal A types is 2.2 years, whereas that of basal type is just 0.5 years [54]. Besides, Luminal subtype cases receiving endocrine and chemotherapy monotherapy, or combined-therapy commonly achieve a long overall survival time of 5 to 10 years [55]. However, basal-like cases present resistance to multiple treatments, whose OS time is commonly less than 5 years [55]. In the present study, the proportion of basal types in high risk group was much higher than that in low risk group (Fig. 2K and Fig. 4N). It suggests that patients with high FR risk score have a greater tendency to be accompanied with the worse-prognosis and treatment-resistant subtype, such as basal type. Regrettably, FR prognostic model could not effectively distinguish the survival differences of basal type patients (Fig. 3O and Fig. 4L), which is a nonnegligible defect.

Except for prognosis, FR risk level also has a tight linkage with tumor immune microenvironment (TIM). As observed above (Fig. 5 and Table 2), the reduced infiltrating levels of CD8+ T and NK cells, which are the main immune effector cells, appeared in high FR risk group. One the other hand, an increment on the immune activity of APC co-stimulation was presented in high FR risk group (Fig. 5H). Considerable evidence indicates that hyperactivation of APC process will lead to immunologic tolerance [45]. It is conceivable that ferroptosis regulatory genes may mediate the formation of immunosuppressive microenvironment and induce immune escape. In fact, iron metabolism extensively participates in the regulation of innate and adaptive immune responses, and there is indeed a crosslink between ferroptosis and TIM [56]. For example, a microarray study has reported that FTL, a gene responsible of encoding the light subunit of the ferritin protein, may be implicated in immune escape and defects of the DNA repair process [57]. In brief, our findings provide some initial clues for elucidating the mechanism of immune tolerance and immune escape in BC. ICIs therapy brings a breakthrough for cancer treatment, especially for metastatic patients. However, only a small fraction of patients can benefit from the promising approach. KEYNOTE-086 study revealed that only 21.4% of TNBC patients present objective responses after receiving pembrolizumab treatment [58]. Unfortunately, through our bioinformatic analyses, FR risk score was weakly associated with the expressions of immune checkpoints. These findings indicated that FR risk may not act as a biomarker for predicting ICIs efficiency.

Recently, cholesterol metabolism is found to play an important role in cancer regulation, and is regarded as a new therapeutic approach [49]. Therefore, SQLE, a key enzyme responsible for cholesterol synthesis, has become a research hotspot gradually. SQLE exerts a complex function in cancer occurrence and development, but commonly serves as a proto-oncogene. In squamous cell lung carcinoma (SCLC), the expression of SQLE was significantly higher in tumor tissues than that in pericarcinoma tissues, and overexpression of SQLE was closely related to poor clinical stages and lymphatic metastasis [59]. In esophageal squamous cell carcinoma (ESCC), SQLE, as a downstream target gene of miR-133b, can induce epithelial-to-mesenchymal transition (EMT) to promoting tumor metastasis [60]. Moreover, prostate cancer patients with high SQLE expression are 8.3 times more likely to have lethal outcomes than that with low SQLE expression [61]. In pancreatic cancer, SQLE plays as a pivotal components of FR prognostic model and is proven to have cancer-promoting functions [62, 63]. In the present study, we confirmed the overexpression of SQLE in mRNA and histological levels, and demonstrated that SQLE was capable of promoting the proliferative, migrative and invasive abilities of BC cells. These findings clarified the biofunctions of SQLE in BC for the first time, and indicated that SQLE may involve in BC progression and hold promise as a potential therapeutic target.

Naturally, this study also has certain limitations. First, the prognostic value of FR risk signature needs further validation in a real clinical cohort. Second, the expression of SQLE is not confirmed through clinical tissues, and its cancer-promoting effects are yet to be demonstrated in vivo. Third, except for SQLE, we have not yet determined the functions of other hub genes in FR risk signature (ALOX15B, ANO6, TP63, JUN, PLIN4, ACSL1 and CHAC1). Therefore, further research is still needed to dissect the roles of ferroptosis regulators in BC.

In conclusions, we constructed a novel FR risk signature. The risk signature was identified as an independent prognostic factor of BC and could increase clinical net benefit. As for immune effect, high FR risk indicated the decreased infiltration levels of NK and CD8+ T cells, whereas promotive that of APCs and their functions. Meanwhile, FR risk score may not serve as a biomarker for predicting ICIs efficacy. Furthermore, we investigated the biofunctions of SQLE in BC cells, which revealed that SQLE possessed the cancer-promoting abilities. In a word, our findings light on future directions for using ferroptosis against breast cancer.

## Supplementary Information

**Additional file 1: Supplementary Fig. 1.** The process of Lasso regression analysis

**Additional file 2: Supplementary Fig. 2.** The immune abundances of 22 leukocyte subtypes in each BC sample. The labels on the X-axis represent the names of each BC sample in TCGA cohort. BC, breast cancer

**Additional file 3: Supplementary Table 1.** The differences in FR gene sets among five research

**Additional file 4: Supplementary Table 2.** The primer lists

**Additional file 5: Supplementary Table 3.** The compositions of different FR risk signatures derived from five research

## Data Availability

The datasets used and/or analyzed in the current study are available from the corresponding author upon reasonable request. All databases in the present study are open, as follows: TCGA database (https://portal.gdc.cancer.gov/), GEO database (https://www.ncbi.nlm.nih.gov/geo/), ICGC database (http://dcc.icgc.org/), METABRIC cohort (http://www.cbioportal.org/), FerrDB database (http://www.zhounan.org/ferrdb/), MSigDB database (https://www.gsea-msigdb.org/gsea/msigdb/) and HPA database (http://www.proteinatlas.org/).

## Abbreviations

BC: Breast cancer
FR: Ferroptosis-related
TCGA: The Cancer Genome Atlas
OSR: Overall survival rate
DCA: Decision curve analysis
NB: Net benefit
ICIs: Immune checkpoint inhibitors
TNBC: Triple-negative breast cancer
MsigDB: Molecular Signatures Database
FRGs: Ferroptosis-related genes
DEGs: Differential expressed genes
ROC: Receiver operating characteristic curve
PCA: Principal component analysis
t-SNE: t-distributed stochastic neighbor embedding
DCA: Decision curve analysis
ssGSEA: Single-sample gene set enrichment analysis
HPA: Human protein atlas
FAs: Fatty acids
TFRC: Transferrin receptors
TF: Transferrin
GPX4: Glutathione peroxidase 4
FTL: Ferritin light chain
FTH1: Ferritin heavy chain 1
PPI: Protein-protein interaction
PD-L1: Programmed cell death 1 ligand 1
CTLA4: Cytotoxic T-lymphocyte associated protein 4
ROS: Reactive oxygen species
CD274: Cluster of differentiation 274
SQLE: Squalene epoxidase
