# Supplementary figures and images for: Ferroptosis regulators, especially SQLE, play an important role in prognosis, progression and immune environment of breast cancer
Source: BMC Cancer. 2021 Oct 29;21:1160. doi: 10.1186/s12885-021-08892-4 (PMC8555209; doi:10.1186/s12885-021-08892-4)

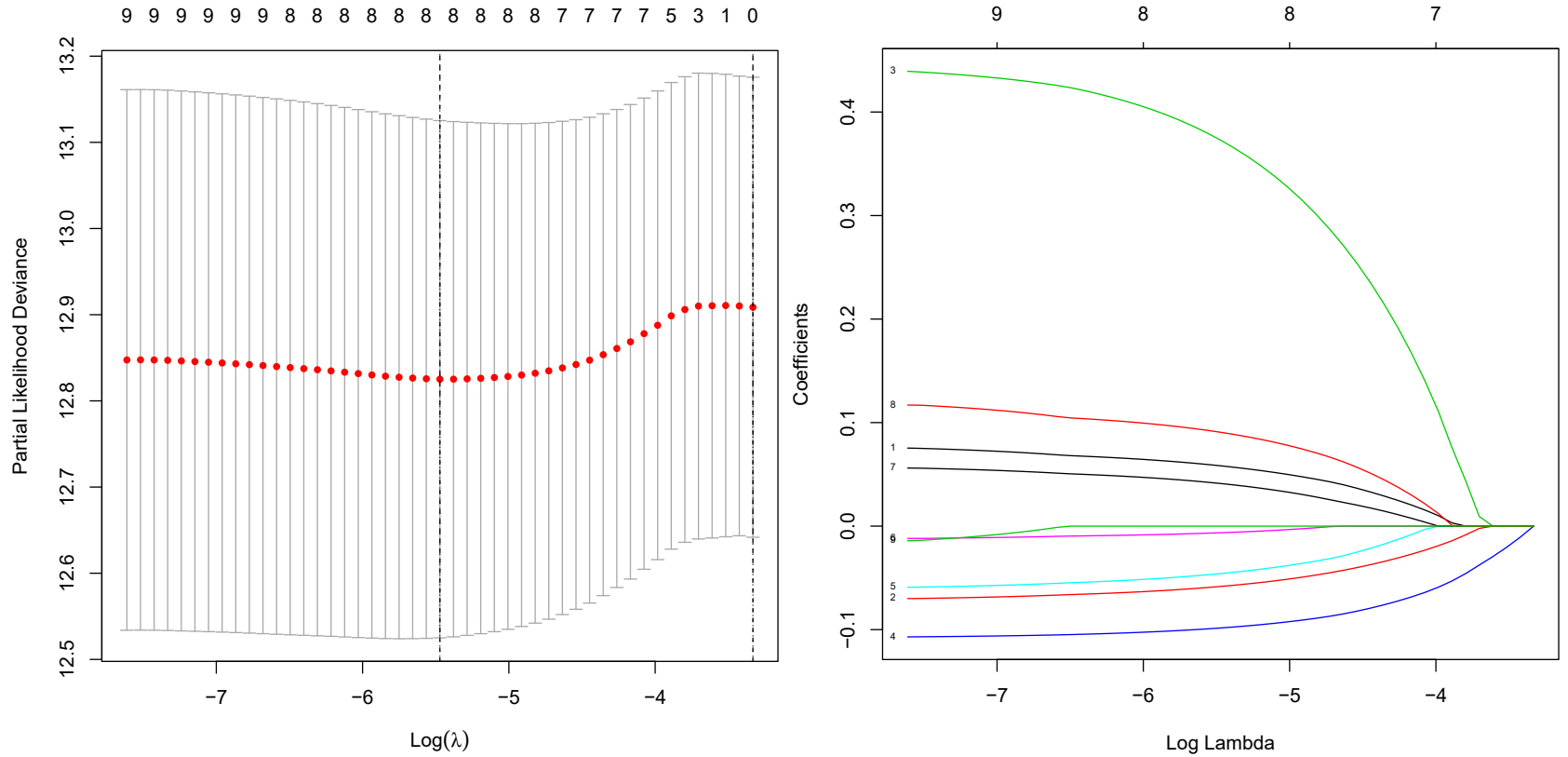

Supplement: Supplementary file 1 — Additional file 1: Supplementary Fig. 1. The process of Lasso regression analysis [file 12885_2021_8892_MOESM1_ESM.jpg]

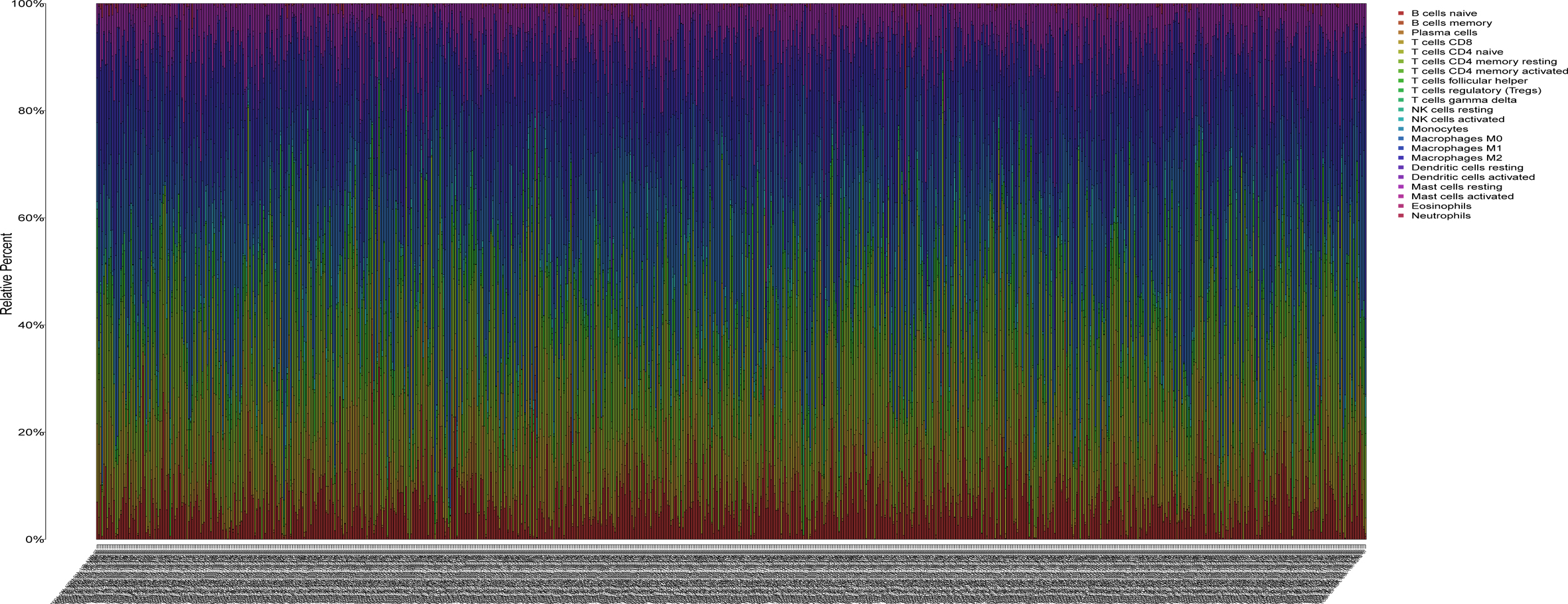

Supplement: Supplementary file 2 — Additional file 2: Supplementary Fig. 2. The immune abundances of 22 leukocyte subtypes in each BC sample. The labels on the X-axis represent the names of each BC sample in TCGA cohort. BC, breast cancer [file 12885_2021_8892_MOESM2_ESM.jpg]
