# Supplementary material for: Ferroptosis regulators, especially SQLE, play an important role in prognosis, progression and immune environment of breast cancer
Source: BMC Cancer. 2021 Oct 29;21:1160. doi: 10.1186/s12885-021-08892-4 (PMC8555209; doi:10.1186/s12885-021-08892-4)
Supplement: Supplementary file 4 — Additional file 4: Supplementary Table 2. The primer lists [file 12885_2021_8892_MOESM4_ESM.docx]

Supplementary Table 2. The primer lists.

| Gene | Primer | Sequence (5' -> 3') |
| --- | --- | --- |
| SQLE | Forward | TGACAATTCTCATCTGAGGTCCA |
|  | Reverse | CAGGGATACCCTTTAGCAGTTTT |
| si-SQLE | Primer | 5'-GCACGAAGAGCCAGTATCAGAAGA-3' |
| pc-SQLE | Forward | GTTAATTAAGGATCCATGTGGACTTTTCTCGGC |
|  | Reverse | GGCCTGCAGGAATTCATGAACCAAGTACTTCATTT |
